# Characterising the unity and diversity of executive functions in a within-subject fMRI study

**DOI:** 10.1038/s41598-022-11433-z

**Published:** 2022-05-17

**Authors:** Rahmi Saylik, Adrian L. Williams, Robin A. Murphy, Andre J. Szameitat

**Affiliations:** 1 Department of Psychology, Mus Alparslan University, Muş, Turkey; 2 Department of Life Sciences, Centre for Cognitive Neuroscience, Brunel University London, Uxbridge, UK; 3 Department of Experimental Psychology, University of Oxford, Oxford, UK; 4 Division of Psychology, Brunel University London, Kingston Lane, Uxbridge, UB8 3PH UK

**Keywords:** Cognitive neuroscience, Learning and memory, Neural circuits, Psychology

## Abstract

Behavioural studies investigating the relationship between Executive Functions (EFs) demonstrated evidence that different EFs are correlated with each other, but also that they are partially independent from each other. Neuroimaging studies investigating such an interrelationship with respect to the functional neuroanatomical correlates are sparse and have revealed inconsistent findings. To address this question, we created four tasks derived from the same basic paradigm, one each for updating, inhibition, switching, and dual-tasking. We assessed brain activity through functional magnetic resonance imaging (fMRI) in twenty-nine participants while they performed the four EF tasks plus control tasks. For the analysis, we first determined the neural correlates of each EF by subtracting the respective control tasks from the EF tasks. We tested for unity in EF tasks by calculating the conjunction across these four “EF-minus-control” contrasts. This identified common areas including left lateral frontal cortices [middle and superior frontal gyrus (BA 6)], medial frontal cortices (BA 8) as well as parietal cortices [inferior and superior parietal lobules (BA 39/7)]. We also observed areas activated by two or three EF tasks only, such as frontoparietal areas [e.g., SFG (BA8) right inferior parietal lobule (BA 40), left precuneus (BA 7)], and subcortical regions [bilateral thalamus (BA 50)]. Finally, we found areas uniquely activated for updating [bilateral MFG (BA 8) and left supramarginal gyrus (BA 39)], inhibition (left IFG BA 46), and dual-tasking [left postcentral gyrus (BA 40)]. These results demonstrate that the functional neuroanatomical correlates of the four investigated EFs show unity as well as diversity.

## Introduction

The central executive system (CES) as a major component of working memory regulates human thoughts and behaviours by maintaining and manipulating information in the storage systems^1,2^. It has been suggested that the CES can be divided into executive sub-functions, such as inhibition, switching, and updating^3^. It is a question of ongoing research whether such Executive Functions (EFs) are distinct from each other (diversity) or whether they rely on a common mechanism or resource (unity). Despite of different views^4–6^, previous behavioural research generally suggested that it is a combination of both particularly in adults^1,7–9^. For instance, Miyake and colleagues^9^ examined the unity and diversity of executive functions by employing various EF tasks in a behavioural experiment. They identified three underlying factors (i.e., three factor model), updating (refreshing and monitoring of mental representations), inhibition (suppression of task irrelevant stimuli that can potentially cause interference) and switching (also called shifting; flexibility in shifting attention between two tasks, operations, or mental sets). These factors correlated with each other, supporting the idea of unity, but also had unique variance, supporting the idea of diversity. Because dual-tasking (simultaneously performing two tasks) was not correlated with these EFs, it has been noted that dual-tasking may constitute an independent EF^9^. Following Miyake and colleagues’ three factor model, dual-tasking was generally ignored and some research suggested that these three EFs may be associated with a common higher-order cognitive control^4,5,10^. Finally, Friedman and colleagues^10–12^ proposed a nested model suggesting that updating and shifting underlies unity and diversity of EFs whereas inhibition has no specific dimension as it is considered as part of shared variance only.

These findings from behavioural studies raise the question about the functional neuroanatomical correlates of executive functions. Do different EFs like updating, inhibition, switching and dual-tasking activate the same or different brain areas? To our knowledge, this important question has been investigated only sparsely, and findings are rather inconsistent. For example, some studies used between-subject designs (i.e., different participant samples for each EF), complicating direct comparisons of brain areas involved in the different EFs^13^. One empirical study by Collette and colleagues^13^ assessed three executive functions, i.e., updating, switching, and inhibition, during PET scanning. The results showed areas of unity in parietal regions (i.e., right intraparietal sulcus and left superior parietal gyrus) and areas of diversity in frontoparietal regions. Although EFs are assumed to be subserved by a fronto-parietal network, it seems a little surprising that no areas of unity were observed in frontal cortices^7^. Potential limitation for this study could be that EF tasks were tested in a different sample (between-subject design) and the tasks were of rather different nature. In more detail, individual differences accompanied by different strategies to accomplish the tasks^13^. Also, in the experimental design using different task domains including various manipulations and S-R (stimuli-response) mappings may affect homogeneity of functions^13–15^.

To circumvent the problems associated with between subject designs, some studies used within-subject designs, i.e., each participant performed several EF tasks. However, previous studies were usually limited to investigating only two EFs at a time. In line with above conclusions, a number of studies associated EFs generally with a frontal-parietal network^15–19^, However, other studies suggested separate localizations for those functions^14,20–22^. Such discrepant findings were not always observed only for different EFs, but also for the same function. For example, when investigating inhibition and switching, Hedden and Gabrieli^15^ observed evidence for unity (i.e., both EFs activated the same areas) while Sylvester and colleagues^22^ found evidence not only for unity, but also for diversity (i.e., areas activated by one EF only). A potential explanation for this discrepancy might be differences in statistical power across the studies or that both studies employed very different task paradigms. Therefore, the current study used highly comparable tasks to assess the four EFs, which were all derived from the same basic paradigm, using the same set of stimuli. Taken together, studies investigating two EFs at a time largely support the idea that the neural correlates of EF may show overlap (unity). However, they are still limited in investigating only two EFs at a time and revealing partially inconsistent findings.

We also reviewed studies that focused on functional neuroanatomical correlates of one single EF only. These studies employed related tasks for a target EF (e.g., n-back for updating, task alternation for switching, Stroop tasks for inhibition, and combination of two simple tasks for dual tasks) generally demonstrate a frontoparietal network for each EFs but also suggest some key regions for exact requirement of the task^23–30^. For instance, a key area for inhibition might be IFG (BA 46) for resolution of conflicts^25–27^, for updating a key area could be the supramarginal gyrus (SMG) as one of main connection channel between frontal and parietal area acting as gate serving to facilitate refreshing of information into WM^23^, for dual-tasking, the areas such as MFG and inferior parietal sulcus (IPS) may particularly involve in task order control^29,30^. Besides of unity areas, activation of such areas in one or more tasks to a different degree may indicate that a natural process of each EF may constitute subprocesses in addition to their unique requirement^31^. For instance, updating involves encoding and selectively maintaining the information in the WM^31^. This process somehow involves the inhibitory process because outdated information is required to be suppressed for the processing of information^31,32^. Likewise dual-tasking may involve the other EFs to some degree because one needs to inhibit the second task until the first task is processed and then switch to the second task^33,34^. Taken together, activation in certain areas can be explained by pairwise correlations of only two EFs, and some variance was unique to each EF.

Finally, metanalysis and review research exploring unity and diversity were generally limited to two or three EFs addressing posterior part of frontal areas and parietal regions for unity and various regions over frontoparietal areas for diversity^35–38^. For instance, Derrfuss and collegues^19,36^ suggested the inferior frontal junction area (IFJ) which located in posterior part of LPFC plays an important role in processing of both switching and inhibition. Another meta-analysis study investigated shared and diverse areas related to dual-tasking and switching addressing bilateral inferior parietal sulcus (IPS) and SPL for unity in addition to distinct areas for each function over fronto-parietal areas^37^. IPL and SPL activation for the unity of EFs also highlighted in a review by Collette and colleagues^38^. These studies demonstrated the evidence that the functional neuroanatomical correlates of EF may mirror the pattern found for the cognitive representations of these EFs, namely that all EFs may activate some common overlapping areas (unity) which included parietal and posterior LPFC, such as left inferior frontal sulcus (IFS), the inferior precentral sulcus (IPrCS)^19,35,36^, and left SPL^13^ but also each activate their unique distinct areas (diversity) such as right IFG for inhibition^26^. However, the exact location of the neural correlates of specific EFs may depend on the exact paradigm used and may differ, at least to some extent, across samples. Therefore, it remains open whether the current pattern of unity and diversity is a mere artifact of a comparison across different studies or whether it reflects the true functional neuroanatomical correlates of EF.

Taken together, previous evidence about the unity and diversity of the neural correlates of different EFs is restricted to studies investigating only one single EF, to studies using between-subject designs, or to within-subject design studies usually investigating no more than two EFs at a time. Therefore, the aim of the current study was to assess the functional neuroanatomical correlates of the four executive functions updating (n-back task), inhibition (Stroop task), switching (task-switching task) and dual-tasking (PRP task) in a within-subject design. To minimise the influence of the tasks, we created four different versions derived from the same basic task, which are based on the same stimuli and mainly differ in the instructions given to the participants. In addition to these key tasks, we also created matching control tasks which are designed to match the perceptual and motor demands and differ only in their demands on the respective EF, so that EF-specific activation can be identified. In detail, the EF of updating was determined by a comparison of a 1-back task with a 0-back task, the EF of inhibition by a comparison of incongruent with congruent Stroop stimuli, the EF of switching by the comparison of a task-switching condition with a task-repetition condition, and finally the EF of dual-tasking by a comparison of dual-tasking with single-tasking (see “Methods” for details). Regarding the analysis, we will use a conjunction analysis to test for overlap of the four EF-specific activation patterns (unity) and pairwise comparisons between the EF-specific activations to test for diversity. These analyses will be supported by detailed analyses of the beta-values obtained from the individual peak coordinates of these contrasts.

## Methods

### Participants

29 participants aged between 18 and 30 (14 Male: mean age 23.50 years, SD 3.32 and 15 Female: mean age 23.50 years, SD 3.32) took part in the experiment. Each participant gave written informed consent and was paid £30 for their participation. We employed the following exclusion criteria: presence of any past or current major medical, neurological or psychiatric illness that might have diminished cognitive functioning; use of psychoactive medication; consumption of alcohol; consumption of ≥ 8 cups or ≥ 900 mg caffeine; scoring over 15 in the Beck depression inventory^39^; colour blindness^40^. The study was approved by the Department of Life Sciences Ethics committee at Brunel University London. The experiment was performed according to relevant guidelines and regulations of the Department of Life Sciences Ethics committee at Brunel University London and declaration of Helsinki.

### Task design and procedure

Participants lay supine in the MRI scanner holding two MRI compatible response pads, wearing MRI compatible in-ear headphones, and viewing a screen via a mirror system. We used a block design to conduct the experiment. There were four main task conditions (EF tasks), and each block of those conditions consisted of 10 trials: updating, inhibition, switching, and dual-tasking. In addition, we added three single task conditions (control tasks), with each block consisting of six trials, and a rest period (baseline), in order to create suitable contrasts^41^. The duration of the blocks varied depending on the condition, with EF blocks lasting 25 s, control task blocks lasting 15 s and the resting baseline lasting 15 s (see also section “Data analysis”). The blocks were presented randomly with ten repetitions of each block. The experiment started with a 15 s baseline block (comprising just a fixation cross) followed by the seven executive and control conditions that were presented randomly with 10 cycles. The experiment ended with one more 15 s baseline block. Each block was preceded by an instruction displayed for 4.5 s and this was followed by a variable number of trials (see below), each lasting 2.5 s. All trials were made up of a 250 ms fixation, 500 ms stimulus, a 2000 ms response interval (starting from stimulus onset) and finally a 250 ms feedback phase which presented an “X” if incorrect and a blank screen otherwise.

We used numbers (1, 2) and colour words (Red, Green, Yellow, Blue) which were presented on the screen with a white background either individually, or in a combination of one number and one word (e.g., 1 Red). The words could either be in a congruent colour (the word Red in red colour) or in an incongruent colour (the word Red in green colour), or in a neutral colour (the word Red in black colour). Participants always responded to the numbers using their left hand, and to the words using their right hand. Based on this, we created various tasks by small variations in the stimuli displayed and the instructions how to respond to them.

#### Control tasks (CT)

For the first control task (CT1), the target stimulus was either the digit ‘1’ or ‘2’. The participant was required to respond as quickly as possible with the left middle finger if the stimulus was ‘1’ or left index finger if it was ‘2’. In the second control task (CT2), the target stimuli were colour names (either ‘Yellow’ or ‘Blue’) presented in their congruent colour and participants had to respond with the right index finger to ‘Yellow’ or the right middle finger to ‘Blue’. In CT3 participants again viewed colour names in congruent colours, but using ‘Red’, ‘Green’, ‘Yellow’ and ‘Blue’. The task required participants to respond with the left middle finger for ‘Red’, the left index finger for ‘Green’, the right index finger for ‘Yellow’, or the right middle finger for ‘Blue’ (i.e., in all conditions, Yellow and Blue were always mapped onto the same fingers). For all three control tasks, six trials were presented in their respective blocks.

#### Executive function tasks

The updating task (UPD) was based on a 1-back task. The stimuli and key mappings were identical to CT3, and participants had to remember and respond to the colour presented in the previous trial. To assess UPD-specific activation, we calculated contrast UPD – CT3 [1], because in this context, CT3 constituted a 0-back condition.

The inhibition task (INH) was based on the Stroop task. The stimuli and key mappings were identical to CT3 except colour names were presented in an incongruent colour (e.g., “Red” written in green). Participants were instructed to respond based on the colour of each stimulus as fast as possible. In other words, they should ignore the written word and focus on the font colour. To assess INH-specific activation, we calculated contrast INH – CT3 [1], because in this context, CT3 constituted a congruent Stroop condition, equivalent to the word reading condition in the traditional paper-and-pencil task.

Dual-tasking (DT) trials presented a combination of digits (as in CT1) and two-colour names (CT2). A single trial might, for instance, present the digit ‘1’ and the colour name ‘Blue’ at the same time, requiring two responses. Participants had to respond first to the digit and then to the colour name, respectively (digit → colour). As two tasks are tapping for the same domain (visual tasks) changing task order would not made a considerable difference, so that we always used same task order. Both stimuli were always presented at the same time, i.e., the stimulus onset asynchrony (SOA) was always 0 ms because we wanted to compare higher executive demand (i.e., 0 SOA dual task) with control tasks which have a negligible executive demand (single tasks). The stimuli were drawn from all combinations of digits and colour names: 1 Yellow, 2 Yellow, 1 Blue, 2 Blue. Ten trials were presented in each block in a random order. Different to all other tasks where one response was required in each trial, the dual-tasking is the only condition where two responses were required per trial. To examine dual-tasking specific activation, in the first level statistics, we calculated the contrast DT − CT1 − CT2, i.e. [1 −1 −1] individually for each participant. The reason for this was that CT1 and CT2 are constituting the single-tasks the dual-task consists of. Therefore, this contrast will reveal dual-tasking specific activations that cannot be explained by summed activation of the single tasks^41^.

In the switching task (SW), the same stimuli as for DT (i.e., “1 Yellow”, “2 Yellow”, “1 Blue”, “2 Blue”) were presented either all in black or in their congruent colour. Therefore, there were 8 stimuli (4 black and 4 coloured version of same stimuli) in this task. If the stimuli were in black, participants had to respond based on the digits displayed (1 or 2) and responded in the same way as described for CT1. If the stimuli were presented in colour, participants had to respond based on the colour (Yellow or Blue) as described for CT2. Because word and colour were congruent, participants could base their decision on the semantics or the ink colour (or both) of the word. The “number” and “colour” cues were presented in a random order so that the participants would switch their responding from one dimension (number or colour) to the other based on the cue. Response mappings for digits and colours and stimuli timing parameters were identical to the dual-tasking. To assess SW-specific activation, we calculated contrast SW – (CT1 + CT2)/2 [1 − 0.5 − 0.5]. Again, CT1 and CT2 constitute the ‘component’ tasks of the SW, but because they only do one task per trial in SW, we subtracted the average.

### MRI procedure

Images of the brain were acquired with a 3 T MRI scanner (Magnetom Trio, Siemens, Erlangen, Germany) equipped with a 32-channel array head coil. 35 axial slices (192 × 192 mm FOV, 64 × 64 matrix, 3 × 3 mm in-plane resolution, 3 mm thickness, no gap, interleaved slice acquisition) were acquired using a BOLD-sensitive gradient echo EPI sequence (TR 2.5 s, TE 31 ms, 85° flip angle). High-resolution whole-brain images were acquired from each participant using a T1-weighted MPRAGE sequence (TR 1900 ms, TE 3.03 ms, 11° flip angle, 176 slices, 256 × 256 mm FOV, 1 × 1 × 1 mm voxel size). A functional run with 755 volumes was acquired, with volume sampling all 35 slices.

### Data analysis

We used SPM12 (www.fil.ion.ucl.ac.uk/spm/) to analyse the MRI data. First, we manually aligned the origin of the structural as well as functional images with the anterior commissure. Head motion was corrected using the Realign & Unwarp option, and all images were transformed into MNI space using normalization and unified segmentation options. Finally, we smoothed the images using a Gaussian kernel with a FWHM of 8 mm. The images were visually checked and validated regarding normalization and registration success. Statistical analysis was based on a voxel-wise least-squares estimation using the general linear model for serially autocorrelated observations^42^. Because the current study used a blocked fMRI design, a boxcar function, convolved with a canonical HRF without derivatives was used to model the BOLD response. To enable comparison across the EF tasks (lasting 25 s) and the control tasks (lasting 15 s), we analysed only the first 15 s of each block for all conditions (in other words, during design specification, the onset was always set on the start of the block, and the duration-parameter was always set to 15 s). A temporal high-pass filter with a cut-off frequency of 1/128 Hz was applied. Individual contrast maps were calculated for all contrasts of interests (see “Results” section), and the second-level analysis was based on one sample t-tests. To test for diversity areas (i.e., the areas showing different activation patterns across the four EFs) in the functional neuroanatomical correlates of the four executive functions, we calculated interaction contrasts between all pairs of executive functions. These interaction contrasts compared whether the EF-specific areas differed from each other by first subtracting the respective control tasks and then comparing the results of these subtractions. For example, to test whether updating and inhibition activated different areas we calculated [(UPD − CT3) – (INH − CT3)] and the reverse [(INH − CT3) – (UPD − CT3)]. To examine common activations across the four executive tasks, we performed a conjunction analysis using logical AND approach^43^. For the conjunction analysis, we employed the contrasts indicated above (see Sect. 2.2.2, e.g., INH − CT3), thresholded at p < 0.05 (FWE corrected). All resulting t-maps were thresholded at a voxel-level p < 0.005 (uncorrected) and only clusters significant at p < 0.05 (FWE corrected) were considered. Anatomical locations and Brodmann areas were determined using the Automated Anatomical Labeling toolbox^44^.

The analyses presented so far cannot easily distinguish between absolute and relative diversity. We use the term *absolute diversity* to indicate that a certain brain area is significantly activated in one EF task only and shows activity virtually not positively different than zero in all other EF tasks. We use the term *relative diversity* to indicate that a certain area is significantly stronger activated in one EF task as compared to other EF tasks, but that there is also significant positive activity in one or more of the other EF tasks as well. Thus, an absolute-diversity area may be considered to be associated with one specific EF but not others, while a relative-diversity may be considered to be associated with more than one EF, but to different degrees. To distinguish between these two types of areas, we first calculated the individual four contrasts to identify activation related to the four respective EFs (e.g. (DT − CT1 − CT2) to identify dual-task related activity and (INH − CT3) to identify inhibition related activity, etc.). Next, we determined areas showing diversity by calculating the above-mentioned interaction contrasts (e.g. (INH − CT3) vs (DT − CT1 − CT2) to test for differences between inhibition and dual-tasking). Finally, we took the peak coordinates as identified in the interaction contrast and checked for activity at this coordinate in the four EF-specific contrasts (e.g. (INH − CT3) for inhibition). For this latter step, we extracted the beta values in a 5 mm sphere around the group peak coordinate, separate for each EF contrast and participant, and tested with a one-sample t-test whether the average beta values differed significantly from 0. If they do so only for one task, then this area is considered to be an absolute-diversity area. If they differ from zero in more than one task, it is considered a relative-diversity area.

## Results

### Behavioural results

**Figure 1:**
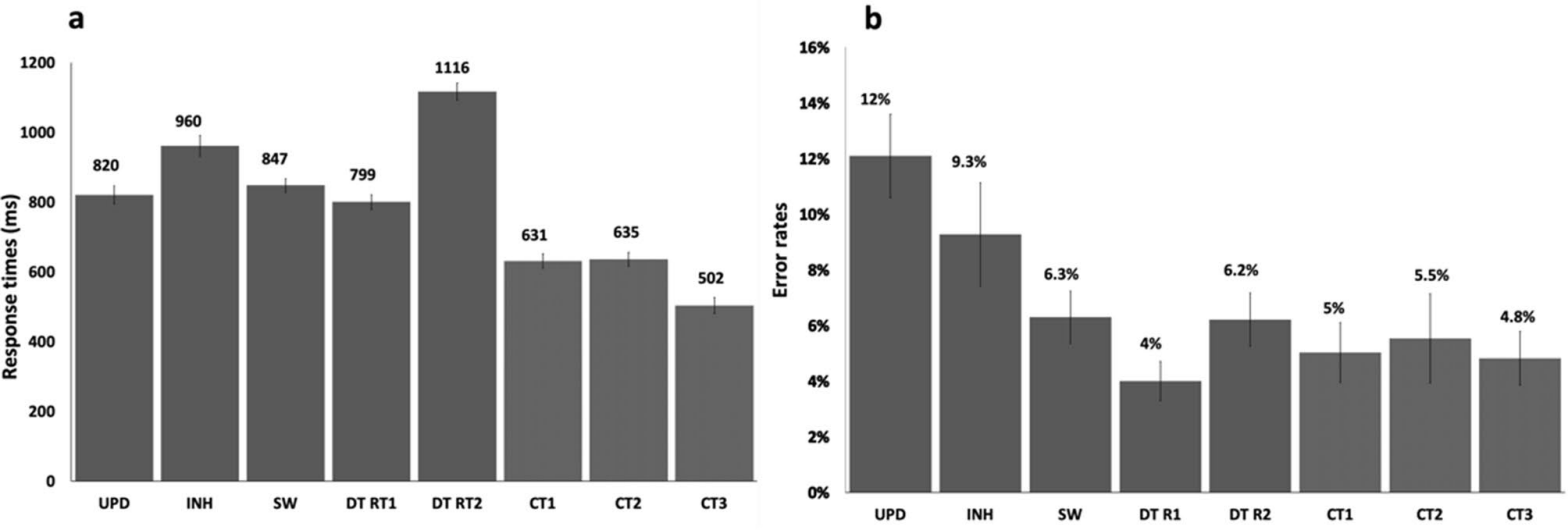
Mean RTs (**a**) and error rates (**b**) for each EF and control task. In the dual-task, the participants always had to respond first to the digits (CT1) and then to the words (CT2), so that dual-task costs were determined as DT response time of the first task (RT1) vs CT1 and DT RT of the second task (RT2) vs CT2. SW was compared with the average of CT1 and CT2. INH and UPD are both compared with CT3, respectively. Error bars denote standard error of the mean (SEM).

We used paired-samples t-tests to examine whether each EF task caused additional behavioural costs when compared to their respective control task(s), e.g., INH vs CT3. Such costs are usually assumed to reflect the EF demanded in the EF task, but not in the control task. For all EFs, we found significantly higher response times (RTs) in the EF tasks as compared to their respective control tasks. In more detail, the results were as follows; for updating (UPD vs CT3; 318 ms; t (28) = 12.24, p < 0.001), for inhibition (INH vs CT3; 458 ms; t (28) = 14.82, p < 0.020), for switching (SW vs average of CT1 and CT2; 214 ms; t (28) = 20.50, p < 0.001) and for dual-tasking (DT-RT1 vs CT1; 168 ms; t (28) = 26.00, p = 0.050 and DT-RT2 vs CT2; 481 ms; t (28) = 26, p < 0.001) (Fig. 1a). We found a similar pattern of results regarding error rates as well, however, the effects were statistically significant only for inhibition (t (28) = 2.97; 4.5%; p = 0.031) and updating (t (28) = 7.94; 7.2%; p < 0.001), while dual-tasking (t (28) = 0.63; 0.7%; p = 0.515) and switching (t (28) = 0.79; 1.1%; p = 0.430) failed to reach significance (Fig. 1b).

### Neuroimaging results

#### Unity: areas commonly activated by all four EFs

To determine the shared neuroanatomical areas activated by all four EF tasks, we first determined the activated areas for each of the four EFs separately using the contrasts (DT − CT1 − CT2) for dual-task related areas, (SW − (CT1 − CT2)/2) for switching-related areas, (UPD − CT3) for updating-related areas, and (INH − CT3) for inhibition-related areas (Supplementary Fig. 1). In a second step, we calculated a conjunction analysis of these four contrasts using the minimum statistics approach^43^.

**Figure 2:**
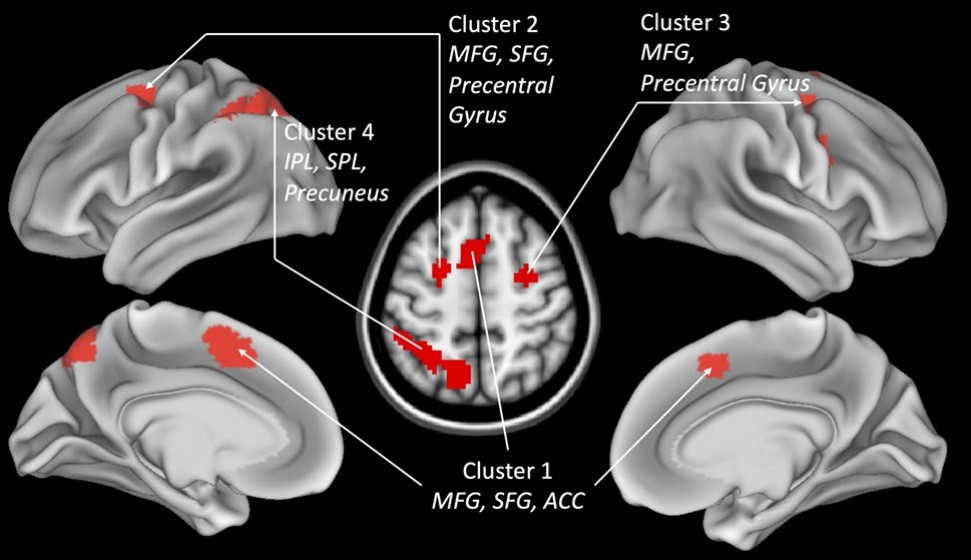
Common activation of the four tasks assessing the executive functions (EF) updating, switching, inhibition, and dual-tasking. To examine common activations across four executive tasks, a conjunction analysis performed with four contrasts UPD-CT3, INH-CT3, SW-average of CT1 and CT2, DT-CT1-CT2 using logical AND approach. Map thresholded at voxel-level p < 0.005 (uncorrected) and cluster-level p < 0.05 (FWE corrected). *MFG* Middle frontal gyrus, *SFG* Superior frontal gyrus, *ACC* Anterior cingulate cortex, *SPL* Superior frontal gyrus, *IPL* Inferior frontal gyrus.

Results (Fig. 2 and Table 1) showed four significant clusters, three in prefrontal cortices and one in the left parietal cortex. The first cluster in the left prefrontal cortex mainly covered an area over medFG (BA 8), partially extending into SFG (BA 6) and anterior cingulate gyrus (ACC, BA 32). The second cluster was localised in the left lateral prefrontal cortex in BA 6 extending into the MFG, SFG and precentral gyrus. The third cluster was localised in the right prefrontal cortex in BA 6, extending into the MFG and precentral gyrus. The fourth cluster was in the parietal cortex extending from the left inferior parietal lobule (IPL, BA 39/40) into the left SPL (BA 7) and reaching around the superior margin of the cerebral hemispheres into the precuneus (BA 7). Therefore, we were able to identify a set of four clusters in a fronto-parietal areas commonly activated by the four different executive functions inhibition, updating, switching, and dual-tasking. This shows that there is unity in the functional neuroanatomical correlates of a diverse set of executive functions.

**Table 1:** Peak activations and anatomical areas of the conjunction analysis (reflecting unity) across all four EF tasks.

Cluster	Anatomical area	BA	X y z	t/p (uncorr)	Cluster-level p(FWE)	Cluster volume (mm^3^)
**1**	medFG	8	− 12 11 47	7.56/0.0001	0.001	920
	SFG	6	-6 7 54			
	ACC	32	-2 6 50			
**2**	MFG	6	-36 -1 53	5.20/0.001	0.04	792
	SFG	6	-23 3 59			
	Precentral gyrus	6	-36 -7 59			
**3**	MFG	6	24 -4 50	3.40/0.01	0.05	392
	Precentral gyrus	6	36 -3 46			
**4**	IPL	39/40	-38 -48 48	6.77/0.001	0.0001	5160
	SPL	7	-27 -59 46			
	Precuneus	7	-9 -65 46			

Thresholded at voxel-level p < 0.005 (uncorrected) and cluster-level p < 0.05 (FWE corrected). *MFG* middle frontal gyrus, *SFG* superior frontal gyrus, *medFG* medial frontal gyrus, *ACC* anterior cingulate gyrus, *IPL* inferior frontal gyrus, *SPL* superior parietal lobe, *BA* Brodmann’s area, *x y z* MNI coordinates.

While the conjunction analysis showed that these four clusters are all activated above threshold in the respective EF-specific contrasts, it might be conceivable that these areas also showed relative differences in activation strength. To test for this possibility, we extracted the beta values from a 5 mm sphere surrounding the peak coordinates in Table 1 from the four EF-specific contrasts (e.g., INH − DT3 for inhibition) for each participant. This provides estimates for the separate EF-specific activation levels at the anatomical locations of unity. Next, we tested for relative differences between the EFs, separate for each peak, by calculating paired-sample t-tests on the extracted beta values (e.g., DT vs INH, DT vs SW, SW vs UPD, etc.). All pairwise comparisons for all peak coordinates were non-significant (all t (28) < 1.33, all p > 0.194), suggesting that there are no significant differences in the activation strengths between the four EFs in the unity areas shown in Table 1 (Supplementary Fig. 2). Therefore, we consider these four clusters as areas of true unity which do not show even relative diversity.

#### Diversity: differences between the four EFs

After having identified areas of unity, we next tested for differences in the functional neuroanatomical correlates of the four EFs. For this, we calculated interaction contrasts for the pairwise comparisons of the four executive functions (Table 2; Supplementary Fig. 3). Note that these interaction contrasts cannot differentiate between absolute and relative diversity and that this distinction will be made further below.

**Table 2:** Peak activations and anatomical areas of the interaction-contrast analyses (reflecting diversity) among all four EF tasks.

Anatomical area	BA	X y z	t/p (uncorr)	Cluster-level p(FWE)	Cluster volume (mm^3^)
**Updating > inhibition (UPD – CT3) – (INH – CT3)**					
SFG	8	27 29 53	5.55/0.0007	0.013	3316
SFG	8	33 17 53			
MFG	8	24 20 41			
**Updating > switching (UPD – CT3) – (SWT – CT1&2)**					
SMG	40	48 − 43 29	6.31/0.00004	0.001	4280
SMG	39	− 45 − 52 26			
MFG	8,9	− 39 17 5	4.81/0.00001	0.001	4440
SFG	6	21 14 50			
SFG	8	30 17 38			
Cingulate gy	31	6 − 49 41	4.24/0.00007	0.01	2848
Precuneus	7	− 3 − 61 62			
**Updating > dual-tasking (UPD – CT3) – (DT – CT1&2)**					
SMG	39	− 48 − 52 32	7.56/0.00001	0.003	3784
IPL	39	− 42 − 67 41			
SMG	39	51 − 46 35	7.49/0.00001	0.001	5016
IPL	39	54 − 52 41			
MFG	8	33 35 44	7.19/0.00001	0.001	13,744
MFG	8	− 42 14 50			
SFG	8	30 29 56			
Cingulate gyrus	31	9 − 43 41	4.32/0.00001	0.001	4928
Para.Cent.L	31	0 − 46 53			
Precuneus	31	− 3 − 43 44			
**Inhibition > dual-tasking (INH – CT3) – (DT – CT1&2)**					
MFG	6	− 42 14 50	5.62/0.00001	0.001	6488
IFG	46	− 48 35 11			
SFG	9,8	3 44 53			
Cuneus	19/18	− 6 − 85 35	5.37/0.00004	0.027	2920
Mid. Temp. Gyrus	19	48 − 76 17			
**Switching > inhibition (SWT – CT1&2) – (INH – CT3)**					
IPL	39	− 34 − 46 58	4.08/00,004	0.013	3768
IPL	40	42 − 34 41			
SPL	7	24 − 58 62			
**Switching > Dual-tasking (SWT – CT1&2) – (DT – CT1&2)**					
Cuneus	18	0 − 97 17	5.60/0.0001	0.017	3040
Cuneus	19	9 − 88 32			
Cuneus	19	− 6 − 88 32			
MFG	6	− 42 14 50	5.36/0.00001	0.001	5488
SFG	8	12 35 59			
SFG	8	− 27 26 56			
CG	31	− 3 − 37 41	5.18/0.00001	0.007	2232
CG	31	6 − 40 41			
**Dual-tasking > switching (DT – CT1&2) – (SWT – CT1&2)**					
Post.cent.L	40	45 − 25 41	8.27/0.00001	0.001	28,416
MFG	6	27 − 7 50			
Post.cent.L	3	− 39 − 28 56			
Thalamus	50	12 − 19 11	6.47/0.0001	0.001	2808
Thalamus	50	− 15 − 19 11			
**Dual-tasking > inhibition (DT – CT1&2) – (INH – CT3)**					
MFG	6	27 − 4 50	7.63/0.00001	0.001	28,200
Post.cent.L	40	39 − 28 41			
**Dual-tasking > updating (DT – CT1&2) – (UPD – CT3)**					
Post.cent.L	6	30 − 10 56	7.83/0.0001	0.0001	10,712
Post.cent.L	40	45 − 25 41			
IPL	40	33 − 37 47			
IPL	40	− 33 − 44 54	7.53/0.0001	0.0001	7560
Post.cent.L	40	− 39 − 28 56			
SFG	6	− 24 − 7 71			

All resulting t-maps were thresholded at a voxel-level p < 0.005 (uncorrected) and only clusters significant at p < 0.05 (FWE corrected) were considered.

##### Updating

A comparison of updating with dual-tasking [(UPD − CT3) − (DT − CT1 − CT2)] showed significant activations in lateral prefrontal, parietal as well as subcortical regions. The cluster in the lateral frontal cortices mainly covered bilateral middle frontal gyri (BA 8) extending into the right superior frontal gyrus (BA 8). The cluster in the parietal cortices covered the left supramarginal gyrus (BA39), extending into the left inferior parietal lobule (BA39). Finally, another cluster in medial parietal regions was mainly located in BA 31 which covered the right posterior cingulate cortex reaching into the paracentral lobule and left precuneus. When updating was compared with switching [(UPD − CT3) – (SW − (CT1 + CT2)/2)], we observed three significant clusters in frontal and parietal areas. The cluster over prefrontal cortices mainly covered left middle frontal gyrus (BA 8/6) reaching into right middle frontal gyrus (BA 6/8) and superior frontal gyrus (BA 6). There were two significant clusters in parietal cortices, one located in right supramarginal gyrus (BA 39/40) and the other in the right posterior cingulate gyrus (BA 31) reaching into the left precuneus (BA 7). Finally, increased activation for updating as compared to inhibition [(UPD − CT3) – (INH − CT3)] revealed a significant cluster in the right lateral prefrontal cortex, extending from the superior frontal (BA 8) gyrus into the middle frontal gyrus (BA 8). Therefore, mainly prefrontal (MFG and SFG) and parietal areas (supramarginal gyrus reaching into posterior cingulate) were more strongly associated with updating than with the other three executive function tasks.

##### Inhibition

A comparison of inhibition with dual-tasking [(INH − CT3) – (DT − CT1 − CT2)] showed significant activations in lateral prefrontal regions extending from the left middle frontal gyrus (BA 6) into left inferior frontal gyrus (BA 46) and right superior frontal gyrus (BA 9/8). A second cluster was located in the bilateral posterior cingulate gyri (BA 31) reaching into the left precuneus (BA 31). A comparison of inhibition with switching and updating revealed no significant activations (p > 0.005, uncorrected).

##### Switching

A comparison of switching with inhibition [(SW – (CT1 + CT2)/2) – (INH – CT3)] showed significant activations in the right inferior (BA 40) and the right superior parietal lobule (BA 7). When compared to dual-tasking [(SW – (CT1 + CT2)/2) – (DT – CT1 – CT2)] the results showed three significant clusters in lateral prefrontal, parietal and occipital cortices. The significant cluster in the lateral prefrontal cortices extended from the left MFG (BA 6) into the bilateral superior prefrontal gyri (BA 8). The cluster in the parietal area was located along the bilateral posterior cingulate gyri (BA 31). Finally, in the occipital cortex significant activations were located in the bilateral cuneus (BA 18/19). There were no significant clusters when comparing switching with updating. Thus, mainly right-hemispheric cortical areas covering superior and inferior parietal lobules as well as bilateral hemispheric cortical areas extending from left MFG to the bilateral SFG reaching into posterior cingulate and cuneus are more strongly associated with switching processing than with inhibition and dual-tasking.

##### Dual-tasking

A comparison of dual-tasking with switching [(DT – CT1 – CT2) – (SW – (CT1 + CT2)/2)] revealed two significant clusters stronger activated for dual-tasking than for switching, one in frontoparietal regions and one in subcortical regions. The large cluster in the frontoparietal region mostly covered the cortical area between the right postcentral gyrus (BA 40) and right middle frontal gyrus (BA 6), but posteriorly also extended into the left postcentral gyrus (BA 3). The cluster in the subcortical region mainly covered the thalamus (BA 50) bilaterally. When dual-tasking was compared with inhibition [(DT – CT1 – CT2) – (INH – CT3)], we observed a similar pattern of results over the frontoparietal region, with the cluster extending from the right postcentral gyrus (BA 40) to the right middle frontal gyrus (BA 6). The results for the comparison between dual-tasking and updating [(DT – CT1 – CT2) – (UPD – CT3)] showed significantly increased activations extending from bilateral postcentral gyri (BA 40) to bilateral inferior parietal lobules (BA 40) and bilateral superior frontal gyri (BA 6). Therefore, mainly right-hemispheric cortical areas extending from the postcentral gyrus and inferior parietal lobe to the middle and superior frontal gyri were more strongly associated with dual-task processing than with the other three executive functions.

**Figure 3:**
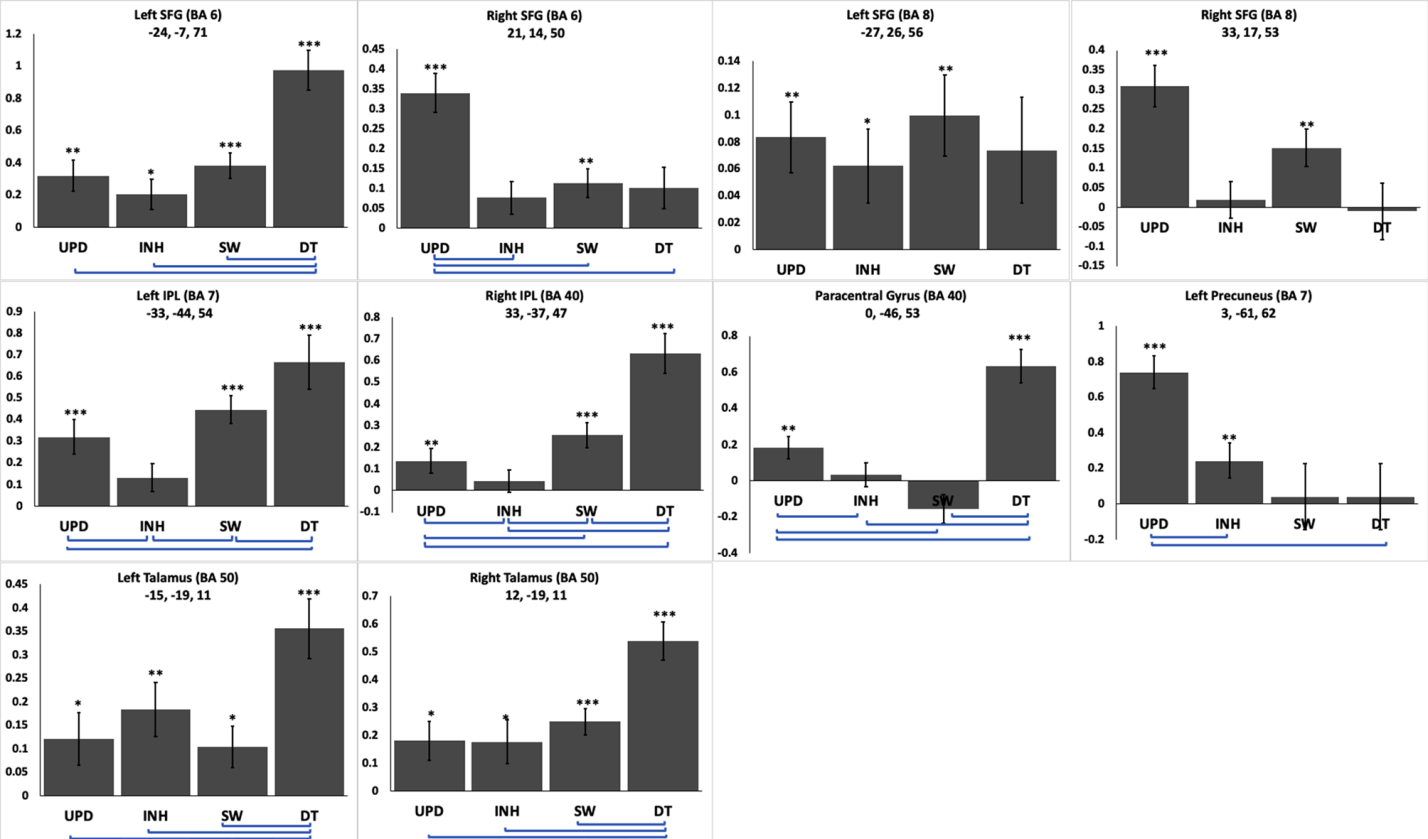
Areas of relative diversity. Each panel corresponds to a coordinate in Table 2. The bars reflect the average beta-values at this coordinate in the respective EF contrasts (e.g., INH – CT3 or DT – CT1 – CT2). Asterisks over each bar indicate significant positive activation in the corresponding task (one-sample t-test vs 0; *p < 0.05; **p < 0.01; ***p < 0.001). Error bars denote SEM. Horizontal lines below the x-axis indicate significant differences between the connected conditions (paired-sample t-tests, p < 0.05). Note the different scales.

**Figure 4:**
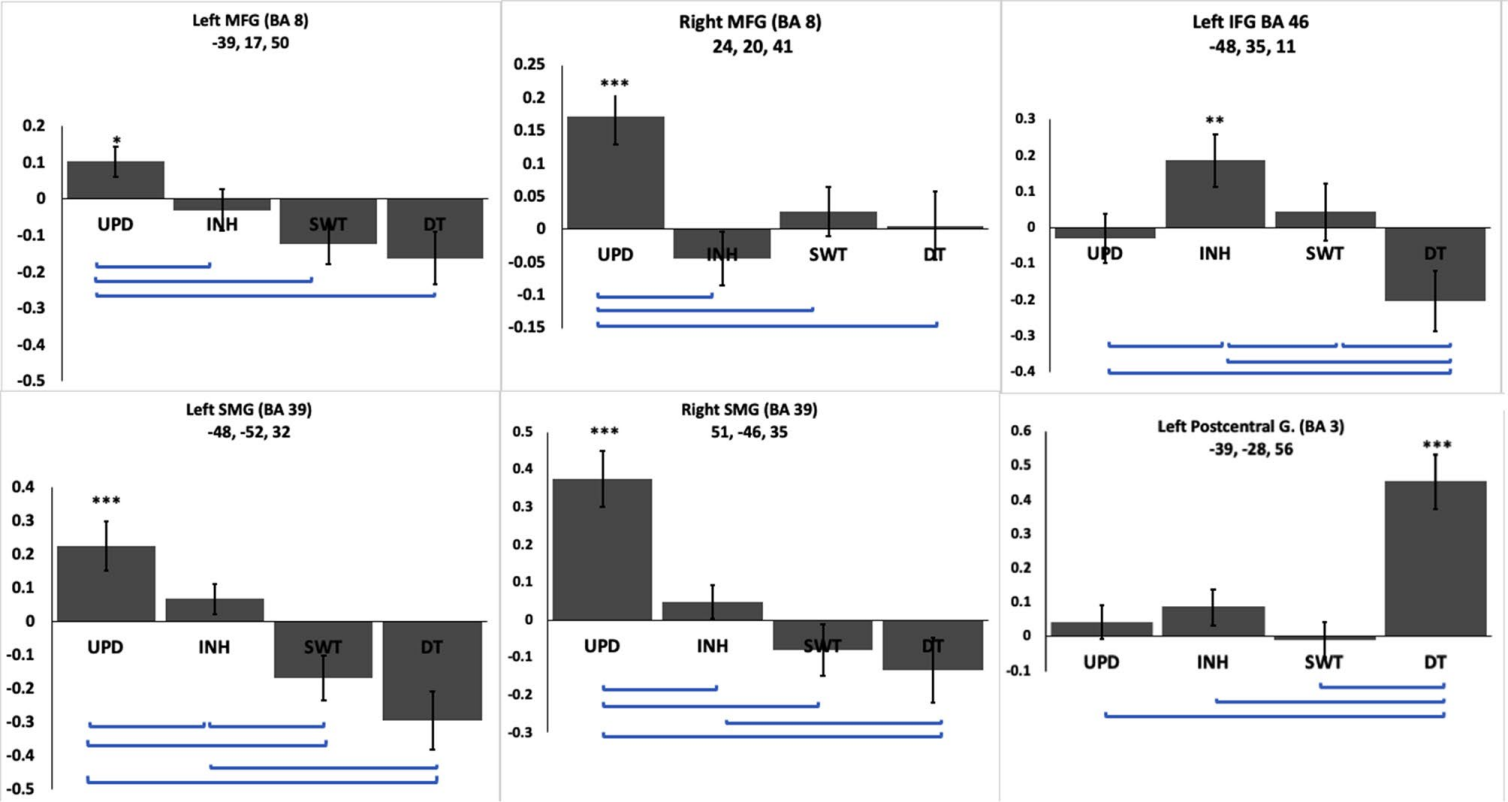
Shown are areas of absolute diversity based on coordinates in Table 2. Asterisks over each bar indicate significant positive activation in the corresponding task *p < 0.05; **p < 0.01; ***p < 0.001. Error bars (SEM) indicate how EFs differentiate from each other. Please note that each graph has a different scale.

Finally, we extracted the beta values in a 5 mm sphere around a selection of the peak coordinates as identified in the interaction contrasts (Table 2) and checked for activity at these coordinates in the four EF-specific contrasts [e.g. (INH – CT3) for inhibition]. Peaks in close vicinity where not individually considered, instead the most significant peak was chosen in Fig. 3. We tested with a one-sample t-test whether the average beta values differed significantly from zero for each EF task. If they differed from zero for more than one task, then we considered this as a relative-diversity area (Fig. 3). If they did so only for one task, then we considered this area as an absolute-diversity area (Fig. 4). Areas showing no activation above zero were excluded.

The results showed that certain areas (i.e., left SFG (BA 6; MNI coordinates x = − 24, y = − 7, z = 71) and bilateral thalamus (BA 50; − 15, − 19, 11 and 12, − 19, 11) were activated for all EFs (all t (28) = t > 2.10, all p < 0.05). We further found that DT activated both areas significantly stronger than UPD, INH and SW, so that we considered these areas as showing relative diversity (all pairwise comparisons t > 5.97, p < 0.001).

Further, there were certain areas showing significantly positive activation only for two or three EFs. Left IPL (BA 7), and right IPL (BA 40) were active only in DT, SW and UPD. Left SFG (BA8) showed similar significant activation for UPD, SW and INH. Right SFG (BA6/8) (UPD and SW) and left precuneus BA7 (UPD and INH) were significantly activated only for two EFs. The right paracentral gyrus showed activation in DT and SW (Fig. 4).

Finally, our results showed areas of absolute diversity, i.e., areas which were significantly activated only in one single EF. In detail, the bilateral MFG (BA 8) and bilateral SMG (BA39) were active only during updating, the left IFG (BA 46) was active only during inhibition, the left postcentral gyrus (BA 40) was active only during dual-tasking, while there were no absolute diversity areas for switching (Fig. 4).

**Figure 5:**
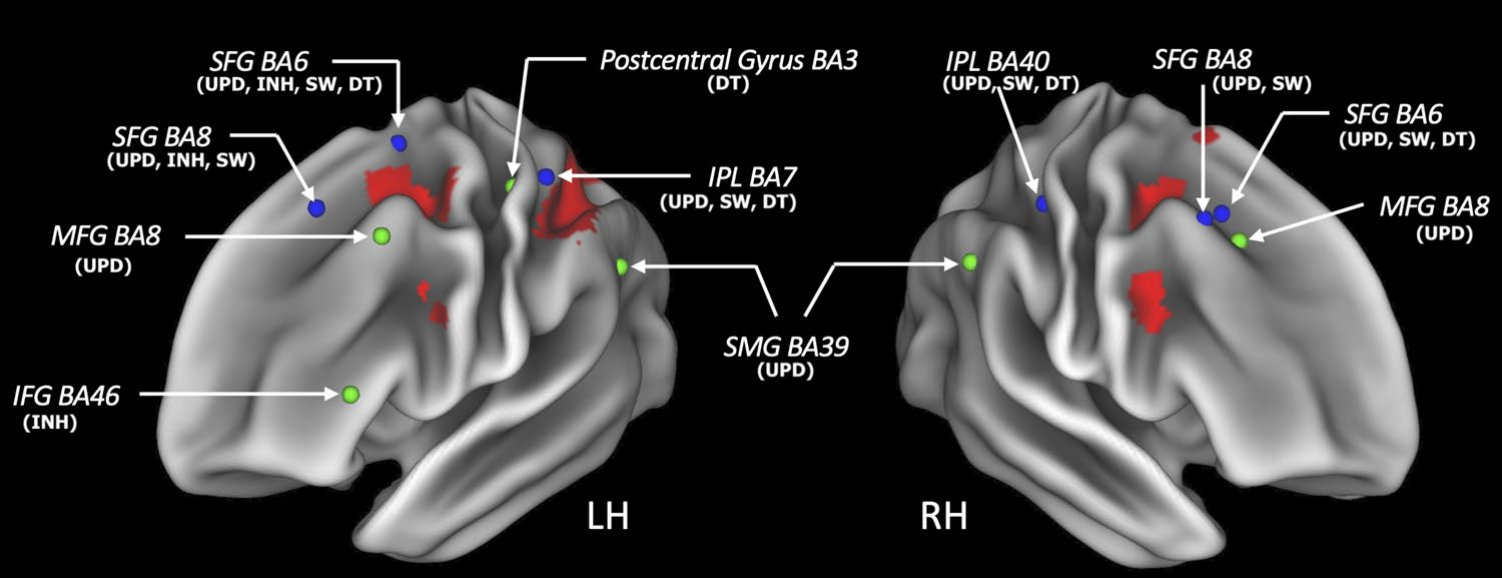
An illustration of areas corresponding to absolute (green spheres) and relative diversity (blue spheres). Unity areas are shown in red and correspond to those shown in Fig. 2. The coordinates of these locations were taken from the regions indicated in Figs. 3 and 4. Note that some coordinates for relative diversity on the medial wall such as thalamus (BA50) and precuneus (BA 7) are not shown here for brevity.

Taken together, we presented an illustration of areas corresponding to relative and absolute diversity in addition to unity areas in Fig. 5.

## Discussion

In this study, we aimed to explore the unity and diversity of the neural correlates of the main executive functions updating, inhibition, switching, and dual-tasking. We developed four target tasks that were derived from the same basic paradigm, one each for updating, inhibition, switching and dual-tasking, and in addition three control tasks. Our behavioural findings demonstrated that participants were slower in each EF task compared to the respective control task. The equivalent analyses in form of the fMRI contrasts (EF tasks – Control tasks) revealed activations in prefrontal and parietal cortices for all four EFs. We then characterised these areas as either showing unity (i.e., areas activated by all four EFs), relative diversity (i.e., areas activated by two or three EFs) or absolute diversity (i.e., areas activated by one single EF only) (Fig. 5).

Unity areas were mainly located in a mostly left-lateralised frontoparietal areas consisting of the medFG, ACC, the precentral gyrus, SFG, and MFG (bilateral), as well as the left inferior and superior parietal lobules and the precuneus. Left hemisphere areas might be more dominant in implementation of EFs based on findings from empirical studies as well as a lesion study. For instance, two empirical studies examined a number of executive functions and both studies generally showed left hemisphere is associated with unity of EFs^13,19^. In line with that, a lesion study demonstrated that patients with left frontal damage performed worse than the ones with right frontal damage in the Tower of Hanoi task that is associated with EFs such as inhibition and planning^45^. In their seminal behavioural study, Miyake and colleagues^9^ identified a shared factor among updating, inhibition and switching reflecting a common mental EF mechanism underlying a variety of EF tasks. We interpret the brain areas showing unity to be associated with this common EF mechanism.

We found only few areas of absolute diversity, i.e., areas which were associated only with one EF but not with any other, which were located in the bilateral MFG (BA 8) and the left supramarginal gyrus (BA 39) for updating, the left IFG (BA 46) for inhibition, and in the left postcentral gyrus (BA 40) for dual-tasking. More areas showed relative diversity, i.e., association with two or three EFs, such as the bilateral SFG (BA 6, 8), right MFG (BA 6), right IPL (BA 40), left SPL (BA 7), paracentral gyrus (BA 40), bilateral thalamus (BA 50), and the precuneus (BA 7). These findings also mirror the correlational patterns among EFs made by Miyake and colleagues^9^ who found that some variance can be explained by pairwise correlations of only two EFs, and that some variance was unique to each EF. Therefore, we interpret these areas of absolute and relative diversity to be associated with mental demands specific to some or even only one EF. Taken together, using a within-subject design we found that the four tested EFs are subserved partially by a common neural basis (unity) and partially by distinct, EF-specific, neural correlates (diversity)^1,9,38^.

We found that the unity areas were organised in the frontoparietal areas, which is consistent with Collette and colleagues^13^ who also reported unity areas in parietal and frontal areas, although the frontal areas were evident only with a reduced threshold and Derrfuss and colleagues^19^ who found a similar pattern of results with current study. In general, a number of studies has shown that EFs are associated with a frontoparietal network^23,35,36^ and that frontal areas (such as the SFG, MFG, and medFG) are functionally connected with parietal areas (such as the IPL and SPL), enabling higher-level mental operations such as implementing goal-directed behaviour, response selection, memory maintenance, and conflict resolution^20,46–48^. Nee and Brown^23^ demonstrated that the posterior part of the left frontal cortex (BA 6) is connected with left posterior parietal areas, and that these frontal and parietal cortices are activated by a wide range of EF tasks. This idea of the frontoparietal areas serving higher-level cognitive operations is consistent with the attentional control view proposed by Engle and colleagues, in which EFs are referring to a generic and domain-free capacity not exclusively linked to specific tasks^49,50^. Taken together, it seems likely that this frontoparietal areas associated with the unity of EFs may act as a central hub of attentional control, integrating input such as representation of task rules from various cortical areas and exerting its control by manipulating neural processing in areas linked to more specific task-related processing^19,47^.

In addition, previous research proposed two potential reasons for the overlap in all three EFs^19,51^. First, a sharing view suggested that certain brain areas may involve in processing of all tasks. This idea of sharing view had been interpreted in two ways. “Reductionist interpretations” states that the shared region could be related to one task and might be borrowed by the other task. That is, a relatively simple task activates an area which is also involved by a more complex task, the activation in the complex task could be reflect a main requirement of the simple task and borrowed by the latter task. The “abstractive interpretation” suggests that shared activities in the brain should be described in more abstractive terms rather than attributing to one task. For instance, the shared areas could be related to more general operations such as monitoring that could be tapped for all EFs. Second, “network view” states that certain brain areas could be activated for all tasks, but this does not necessarily mean that it associates with the same cognitive operation because the shared areas may be functionally linked with the other brain areas that may help to run different operations. It has been indicated that these views are not conflicted they rather could be combined in different ways. In this regard, it seems likely that abstractive interpretation is more consistent with our findings regarding unity of the four EFs. The reason for this is that to minimise the influence of the tasks, we created four different versions derived from the same basic task, which are based on the same stimuli and mainly differ in the instructions given to the participants. As the reductionist interpretation usually used for overlapping between two or three tasks, we adopt it for interpretation of relative diversity below.

In the current study, we found unity in the neural basis of EFs not only for switching, inhibition and updating but also for dual-tasking. Miyake and colleagues^9^ suggested that three EF tasks (i.e., switching, inhibition and updating) correlated with each other in terms of behavioural performance, but did not observe correlations with dual-tasking. While Miyake and colleagues^9^ suggested that dual-task processing may demand an independent executive function, our neuroimaging findings suggest that dual-tasking shares the neural correlates with the other three EFs. A potential reason for this discrepancy might be the use of different dual-task paradigms. Miyake and colleagues^9^ used a rather complex dual-task paradigm composed of two different working memory tasks (Maze Tracing Speed test and letter generation) for which the exact temporal structure of the mental processes is hard to establish. In the current task, we combined two speeded choice-response tasks presented simultaneously, for which it is well known that both tasks compete for a limited processing resource^52^. Our finding that dual-tasking also activated the unity areas are in agreement with behavioural studies^53,54^, theoretical models^55,56^ as well as neuroimaging studies^33,57,58^ suggesting that dual-tasking demands EFs to coordinate the concurrent processing of two tasks. Thus, our study suggests there is unity in the neural correlates of four different EFs.

The relative diversity areas showed that EFs commonly activated certain areas in both hemispheres with varied strengths. In line with reductionist view, this observation may indicate that certain EFs may partially overlap to some degree in their mental demands through common task requirements^19,51^. For example, it is conceivable that most switching tasks also demand inhibition, because for a successful switching operation the task to be switched away from might need to be inhibited^15,19,23^. Likewise, a number of studies agreed that dual-task processing involves inhibition, switching and updating^58,59^. Taken together, the diversity areas may be related to these specific task requirements. While some of these specific requirements may partially overlap, others might be unique^31,51^.

We found areas of absolute diversity, i.e., areas which were significantly activated by only one single EF, for inhibition, updating and dual-tasking. These areas are consistent with previous research linking them to the corresponding EFs. For instance, the IFG has been repeatedly found to be a key area of inhibition^26,60^ and dual-tasking has been shown to be impaired when the left postcentral gyrus (BA 40) is lesioned^61^. Finally, updating has been linked to the bilateral MFG (BA 8) and left SMG (BA 39)^24,62^, potentially by demands on active monitoring^31^. We did not find an area of absolute diversity for switching. Whether this is caused by a lack of statistical power, by the particularities of the task, or whether switching is not associated with any unique areas cannot be answered in the current study.

We would like to point out that our discussions of relative and absolute diversity refer to the four EFs investigated in this study. We do not rule out that those areas are activated by other EFs not investigated here, or even other mental processes not linked to executive functions. In other words, we do not consider them to be exclusively associated with the here investigated functions. Consequently, an area which we considered to show absolute diversity because it was active in only one single EF in this study might be activated by another EF, e.g., planning, and may then be considered as an area of relative diversity.

In the analysis for relative diversity in which we analysed the beta-values of all four EFs we identified areas to be activated by all four EFs, although they were not identified in the conjunction analysis testing specifically for unity (e.g., thalamus (BA 50) for dual-tasking). However, the analysis of beta-values was more sensitive (uncorrected p < 0.05) as compared to the conjunction analysis (corrected cluster-level p < 0.05). The reasoning behind the use of different thresholds was that in the analysis of beta-values we wanted to ensure that we do not misidentify an area as absolute diversity although it may show some activity in other areas (i.e., we aimed to rule out beta-errors). On the other hand, in the conjunction analysis we wanted to ensure that we only identify areas which were truly activated by the four EFs (i.e., we aimed to rule out alpha-errors). Therefore, the use of different thresholds can explain such observations. In general, the exact spatial extend of activations depends on the chosen thresholds. Thus, in particular at the edges of the activation clusters, where unity areas may smoothly transition into relative diversity areas, the exact locations of these transitions are likely to be influenced by statistical power and the statistical thresholds. Therefore, caution is advised when interpreting the exact extent of unity areas and relative and absolute diversity areas.

The current study has some inherent limitations that should be considered in future research. First, block length for EF tasks (25 s) and control tasks (15 s) were different due to an unwitting error during experimental design. The analysis presented here was taken from the first 15 s for each block (see “Methods”), but we also ran an identical analysis using the full block lengths and this revealed a very similar pattern of results. Second, tasks within each block were presented in a repeated (but randomised) fashion. It is well established that task repetition may lead to habituation effects as participants get used to the task. We examined task response times, and overall, there seemed to be no significant change in RTs across trials. On this basis, we believe it is unlikely that there are habituation effects within our data, but future studies should consider this possibility. Thirdly, our dual-task specified that participants had to respond in a specific order, rather than have the autonomy to respond in whichever order they prefer^63^. This may invoke brain regions different to those associated with dual-tasking without any required order, for example, by introducing additional working memory demands. Lastly, the analysis of our neuroimaging data used a standard smoothing kernel size which invariably has implications for the identification and interpretation of activity patterns (as filter width increases, the spatial extent of activation becomes larger). Whether the brain regions identified here associated with unity and diversity are sensitive to the kernel size is unclear but given the relative proximity of some of these regions, this cannot be ruled out.

In conclusion, the current study aimed to explore the neural correlates of four executive functions: switching, inhibition, updating and dual-tasking. We found that certain areas in frontoparietal cortices were commonly activated by all four EFs and suggest that these areas are associated with a unitary core function such as controlled attention. We interpret areas which were activated by one, two, or three EFs as being associated with more specific mental demands linked to some EFs only. Our data show that the previously suggested organisation of EFs into unity and diversity is also reflected in their functional neuroanatomical correlates.

## Supplementary Information

Supplementary Figures.

## Data Availability

The stimuli and processed data files are available at 10.6084/m9.figshare.19780264.
