# Author Correction: Characterising the unity and diversity of executive functions in a within-subject fMRI study

**DOI:** 10.1038/s41598-022-14565-4

**Published:** 2022-06-14

**Authors:** Rahmi Saylik, Adrian L. Williams, Robin A. Murphy, Andre J. Szameitat

**Affiliations:** 1 Department of Psychology, Mus Alparslan University, Muş, Turkey; 2 Department of Life Sciences, Centre for Cognitive Neuroscience, Brunel University London, Uxbridge, UK; 3 Department of Experimental Psychology, University of Oxford, Oxford, UK; 4 Division of Psychology, Brunel University London, Kingston Lane, Uxbridge, UB83PH UK

Correction to: *Scientific Reports* 10.1038/s41598-022-11433-z, published online 17 May 2022

The original version of this Article contained an error in the Data Availability section where

“The stimuli and processed data files will be available available at: figshare.”

now reads:

“The stimuli and processed data files are available at https://doi.org/10.6084/m9.figshare.19780264.”

The original Article has been corrected.

