# Supplementary material for: Characterising the unity and diversity of executive functions in a within-subject fMRI study
Source: Sci Rep. 2022 May 17;12:8182. doi: 10.1038/s41598-022-11433-z (PMC9114123; doi:10.1038/s41598-022-11433-z)
Supplement: Supplementary file 1 — Supplementary Figures. [file 41598_2022_11433_MOESM1_ESM.docx]

**SUPPLEMENTARY MATERIALS**

**Characterising the unity and diversity of executive functions in a within-subject fMRI study**

**Rahmi Saylik^1,2^ Adrian L Williams^2^ Robin A Murphy^3^ Andre J Szameitat^2^**

^1^Mus Alparslan University, Department of Psychology,

^2^Brunel University London, Department of Life Sciences

^3^University of Oxford, Department of Experimental Psychology

**The unity imaging contrasts (Supplementary material figure 1)**


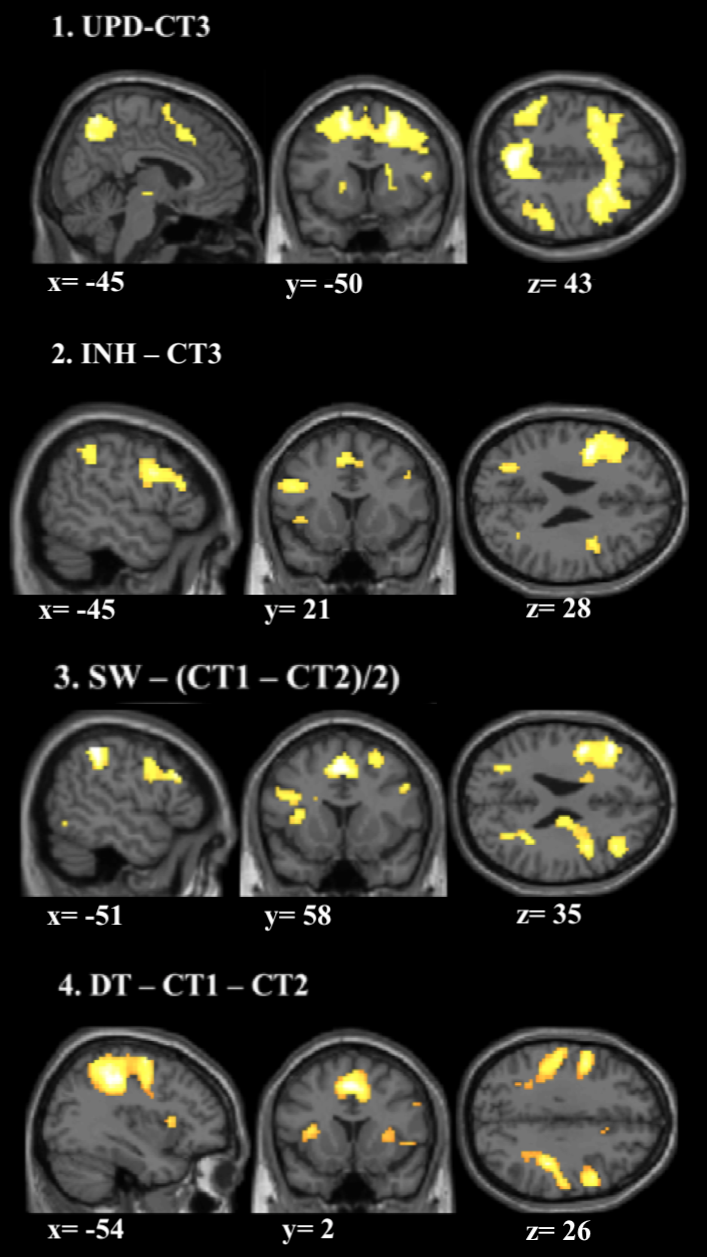


Supplementary material figure 1. imaging data. To examine common activations across four executive tasks, a conjunction analysis performed with four contrasts (1) UPD-CT3, (2) INH-CT3, (3) SW-average of CT1 and CT2, (4) DT-CT1-CT2. Map thresholded at voxel-level p < 0.005 (uncorrected) and cluster-level p<0.05 (FWE corrected).

**The unity graphs (supplementary figures 2)**

**
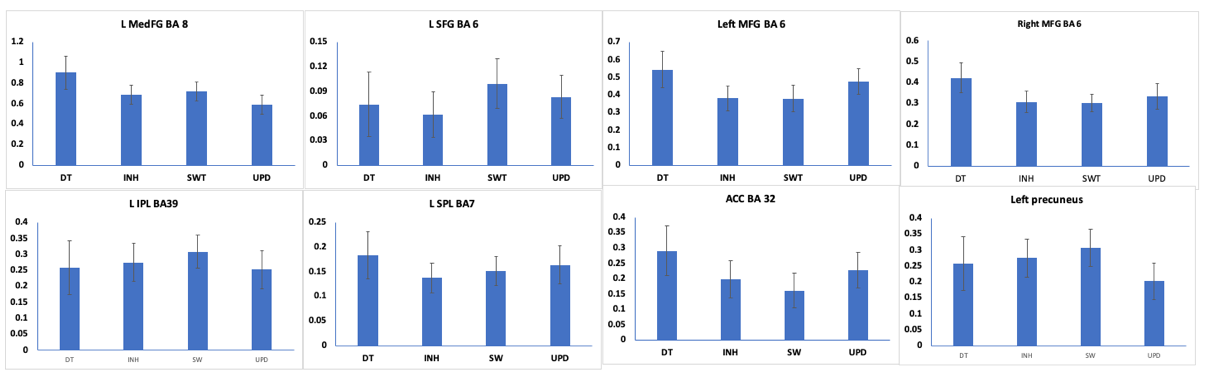
**

Supplementary materials figure 2. Areas of unity. Each panel corresponds to a coordinate in Table 1. The bars reflect the average beta-values at this coordinate in the respective EF contrasts (e.g., INH – CT3 or DT – CT1 – CT2). Error bars denote SEM. Note the different scales.

**The interaction imaging contrasts (Supplementary material figure 3)**


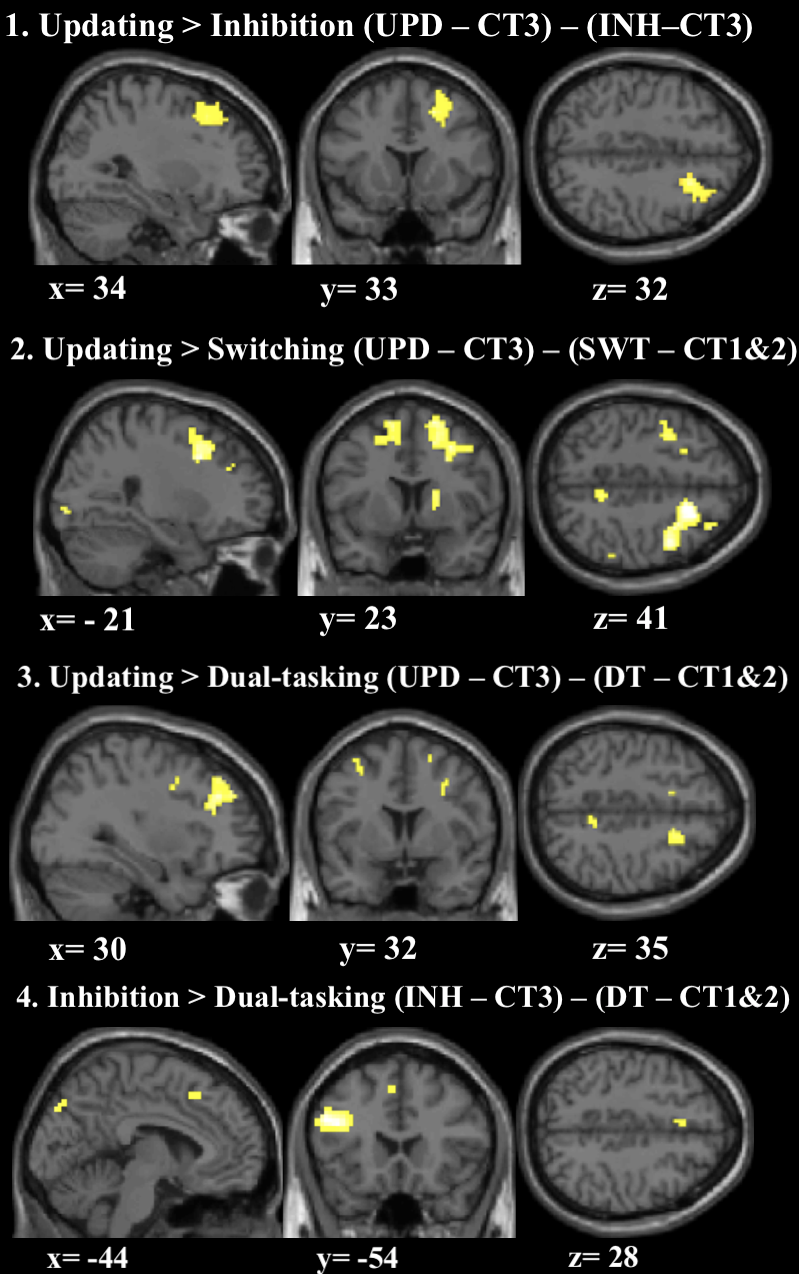


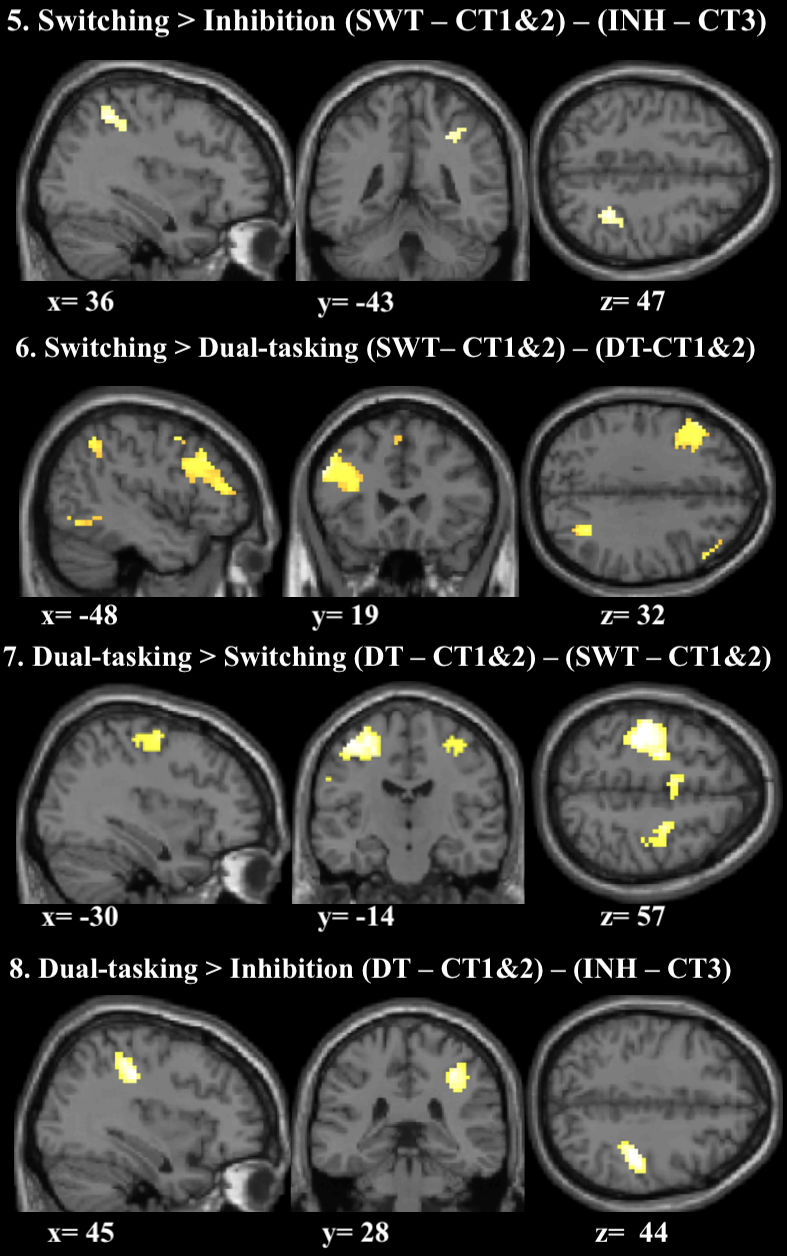


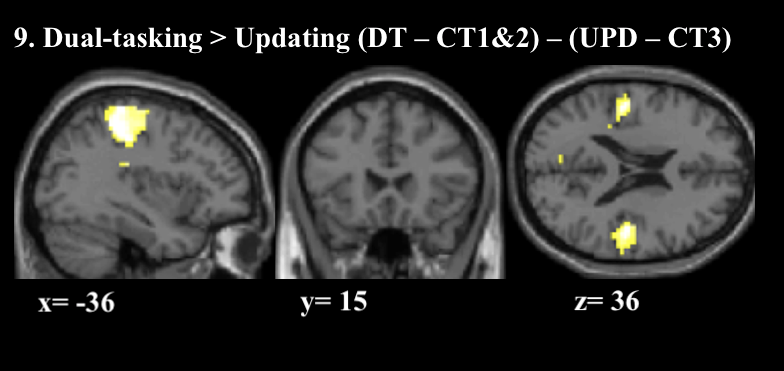


Supplementary material figure 3. Anatomical areas of the interaction-contrast analyses (reflecting diversity) among all four EF tasks (image numbers 1-9). All resulting t-maps were thresholded at a voxel-level p < 0.005 (uncorrected) and only clusters significant at p<0.05 (FWE corrected) were considered.
